# Is MRA an unnecessary expense in the management of a clinically unstable shoulder? A comparison of MRA and arthroscopic findings in 90 patients

**DOI:** 10.3109/17453674.2012.672090

**Published:** 2012-06-04

**Authors:** Sam C Jonas, Michael J Walton, Partha P Sarangi

**Affiliations:** ^1^Department of Orthopaedics, University Hospital Bristol; ^2^Avon Orthopaedic Centre, Southmead Hospital, Bristol, UK

## Abstract

**Background and purpose:**

In detection of glenoid labrum pathology, MR arthrography (MRA) has shown sensitivities of 88-100% and specificities of 89-93%. However, our practice suggested that there may be a higher frequency of falsely negative reports. We assessed the accuracy of this costly modality in practice.

**Patients and methods:**

We retrospectively reviewed MRA reports of 90 consecutive patients with clinical shoulder instability who had undergone shoulder arthroscopy. All had a history of traumatic anterior shoulder dislocation and had positive anterior apprehension tests. All underwent arthroscopy and stabilization during the same procedure. We compared the findings, using arthroscopic findings as the gold standard in the identification of glenoid labrum pathology.

**Results:**

83 of the 90 patients had glenoid labrum tears at arthroscopy. Only 54 were correctly identified at MRA. All normal glenoid labra were identified at MRA. This gave a sensitivity of 65% and a specificity of 100% in identification of all types of glenoid labrum tear. 74 patients had anterior glenoid labral tears that were detected at an even lower rate of sensitivity (58%).

**Interpretation:**

The sensitivity of MRA in this series was substantially lower than previously published, suggesting that MRA may not be as reliable a diagnostic imaging modality in glenohumeral instability as previously thought. Our findings highlight the importance of an accurate history and clinical examination in the management of glenohumeral instability. The need for MRA may not be as high as is currently believed.

Bankart (1923) first described damage to the anterior glenoid labrum as being a cause of recurrent anterior dislocation. Labral lesions are known to lead to higher rates of instability. Open surgical stabilization is effective, and modern arthroscopic techniques with suture anchors have similar success rates (Hobby et al. 2007). Arthroscopic techniques have the advantage of not violating the subscapularis tendon and allow a diagnostic evaluation of the joint and capsulo-lig mentous structures prior to reconstructive surgery.

Various imaging modalities have been used to identify glenoid labral lesions, including arthrography, CT arthrography, MRI, and MRI arthrography (MRA). MRA has proven to be the most sensitive (Chandnani et al. 1993, Palmer et al. 1994). Studies evaluating the sensitivity of MRA in the detection of glenoid labral lesions, using arthroscopy as the gold standard, have found high sensitivity (88–100%) and high specificity (91–93%) (Flannigan et al. 1990, Chandnani et al. 1993, Palmer et al. 1994, Palmer and Caslowitz 1995, Tirman et al. 1997, Waldt et al. 2005, Holzapfel et al. 2010, Iqbal et al. 2010).

MRA is, however, more invasive than conventional MRI. This has implications such as increased cost, longer waiting list times, and an increased number of potential risks associated with the procedure (Newberg et al. 1985). Normal variations in anatomy may also reduce sensitivity (Beltran et al. 1997).

The high reported accuracy of standard 1.5-T MRA has lead to it being integral to the pathway for many patients with anterior instability, possibly at the expense of an accurate history and clinical examination by a specialist shoulder surgeon. We have therefore assessed the accuracy of MRA in a group of patients undergoing anterior stabilization for clinical instability. Our hypothesis was that 1.5-T MR arthrography is not as sensitive as previously believed.

## Patients and methods

90 consecutive patients (78 men) undergoing arthroscopic anterior stabilization surgery were identified over a 3-year period. Mean age was 27 (15–53) years. All patients had a history of traumatic anterior shoulder dislocation with persistent symptoms of instability. 41 had dislocated twice or less, 47 had dislocated more than twice, and 2 had a history of persistent subluxation. At clinical examination, all patients had a positive anterior apprehension test. They all had a preoperative MRA; this was thought to help in surgical planning.

52 of 90 arthrograms were performed and interpreted by 4 consultant radiologists with more than 10 years of musculoskeletal experience. The remainder were performed and interpreted by 3 other consultant radiologists with varying musculoskeletal experience. Under fluoroscopic guidance, using local anesthetic, 14 mL of iodinated contrast (containing dilute gadolinium: 1:200) was injected into the glenohumeral joint. Using dedicated shoulder coils, the following sequences were obtained on a Siemens Avanto 1.5 Tesla MRI scanner: axial T1 weighted gradient echo (high resolution, thin slice thickness), axial TSE T1 weighted, sagittal oblique and coronal oblique fat-suppressed SE T1 weighted, and coronal oblique TSE T2 weighted images. The performing radiologist then interpreted the MRI arthrograms. The criterion used for the detection of glenoid labral pathology was that of an obvious change in morphology or detachment visualized in the labrum, or a change in signal intensity that would be consistent with such a change. Only 1 radiologist interpreted each MR arthrogram.

We performed an examination under anesthesia in all cases before surgery. Arthroscopy was performed in the lateral decubitus position with longitudinal traction. A posterior soft-spot portal was created and a diagnostic arthroscopic evaluation of the glenohumeral joint was performed with probing of the glenoid labrum via an anterior portal. Stabilization procedures, including anterior labral repair and capsular imbrication, were performed as required. A single consultant orthopedic shoulder surgeon with more than than 10 years of experience performed all procedures.

This work was registered and approved by the North Bristol NHS Trust clinical audit department (registration number 1414).

### Statistics

Sensitivities, specificities, positive and negative predictive values, and their respective 95% confidence intervals (CIs) were calculated in the detection of glenoid labrum pathology using SPSS software version 16.

## Results (Table)

83 of the 90 patients had a glenoid labrum tear identified at arthroscopy. These were all described as having substantial labral damage, 74 with avulsion of the anteroinferior labrum consistent with a Bankart lesion, 4 with avulsion of the posterior labrum, and 7 with avulsion of the superior anterior to posterior labrum consistent with a SLAP lesion. Of the remaining 7 cases, 2 were described as having a mobile labrum with a degree of anterior capsular laxity but no labral detachment, 3 were described as cases of medial subluxation, and 2 were described as having an essentially normal but anteriorly scuffed labrum. All patients underwent arthroscopic stabilization of the shoulder.

**Table:** Sensitivity and specificity of MRA with arthroscopy findings as gold standard

Finding at arthroscopy	No. found at MRA	No. found at arthroscopy	True positive	True negative	False positive	False negative	Sensitivity of MRA	Specificity of MRA
Anterior labral tear	43	74	43	16	0	31	0.58 (0.47–0.69)	1.00 (0.81–1.00)
Posterior labral tear	4	4	3	85	1	1	0.75 (0.30–0.95)	0.98 (0.94–0.99)
Slap lesion	7	5	5	83	2	0	1.00 (0.56–1.00)	0.97 (0.91–0.99)
Any labral tear	54	83	54	7	0	29	0.65 (0.54–0.74)	1.00 (0.65–1.00)
Normal labrum	36	7	7	54	29	0	1.00 (0.65–1.00)	0.65 (0.54–0.74)

MRA allowed correct identification of the 2 normal labra, the 3 cases of medial labral subluxation, and the 2 cases of capsular laxity. Of the 83 cases with labral avulsion, 43 were correctly identified as having anterior tears, 3 were correctly identified as having posterior tears, and 5 were correctly identified as having SLAP lesions. 1 was reported as having a posterior tear and 2 were reported as having a SLAP lesion, all 3 of which had anterior tears at arthroscopy. 29 patients were reported as having a normal labrum: 28 had an anterior tear and 1 had a posterior tear.

Overall 54 of 83 patients with some sort of labral pathology were identified at MRA, giving a sensitivity of 65% (CI: 0.54–0.74) and a specificity of 100% (CI: 0.65–1.00). Most patients (74 of 83) with labral pathology were found to have anterior tears. Of these, 43 were correctly identified at MRA, giving a sensitivity of 58.1% (CI: 0.47–0.69) and a specificity of 100% (CI: 0.81–1.00) in detection of anterior labral tears.

## Discussion

Although several studies have examined the sensitivity of the MR arthrogram in detecting glenoid labral lesions, the majority had small sample size and identified the patient group at the time of arthroscopy—retrospectively evaluating those with proven labral lesions rather than prospectively including all clinically unstable shoulders that were indicated for surgery (Chandnani et al. 1993, Palmer et al. 1994, Waldt et al. 2005).

Palmer and Caslowitz (1995) and Waldt et al. (2005) are currently the largest published studies (n = 121 and n = 104). The subjects were identified at arthroscopy and the MRAs were reviewed retrospectively. Waldt et al. showed sensitivities of 88% for detection of labro-ligamentous damage and 77% for correct diagnosis of specific lesion types. Palmer and Caslowitz showed sensitivity and specificity of 92% for identification of labral lesions. Other, smaller studies have had sensitivities ranging from 91% to 100% (Flannigan et al. 1990, Chandnani et al. 1993, Palmer et al. 1994, Waldt et al. 2005, Holzapfel et al. 2010, Iqbal et al. 2010). These smaller studies included subjects with shoulder instability similar to what we describe here, but several used other indicators for inclusion such as shoulder pain, which we did not use.

Our aim was to assess the sensitivity of the preoperative MRA in those patients who were indicated for arthroscopic stabilization on clinical grounds. We found substantially lower sensitivity (65%) in detection of labro-ligamentous damage found at arthroscopy than has been reported in other published studies (Flannigan et al. 1990, Chandnani et al. 1993, Palmer et al. 1994, Palmer and Caslowitz 1995, Tirman et al. 1997, Waldt et al. 2005). This difference may be explained by the fact that the 2 largest published studies (Palmer and Caslowitz 1995, Waldt et al. 2005) were performed by experienced and specialized musculoskeletal radiologists. The reliability of interpretation of MRA may be more accurate in their hands. However, our series—and most surgeons' practices—rely on MRAs being reported by many different radiologists of varied experience and expertise. This lower sensitivity may be a more accurate reflection of routine clinical practice.

With a specificity of 100%, the presence of a normal MRA in conjunction with a normal examination would appear to be reassuring confirmation of one's clinical acumen. However, bearing in mind the sensitivity (65%), a “normal” MRA should not be used as a rationale for not operating on a symptomatic individual. Most of our patients had anterior labral tears. Thus, the results may not be applicable to the interpretation of MRA in detection of other types of glenoid lesions.

Recent studies by Magee and Williams (2006) and Magee (2009) have shown that 3-T MR arthrograms may have higher levels of sensitivity in the detection of glenoid labrum lesions (95–98%) than those using 1.5-T, which is the general convention. This would make this modality a much more attractive diagnostic tool. However, until such MRAs are in general use, we should consider 1.5-T MRA as our everyday standard.

Although our study has a similar sample size to those reported previously, it has several shortcomings that could not be altered due to the retrospective nature of the study design. All the arthroscopies were performed by a specialist shoulder surgeon, but the MR arthrograms were performed by 7 different radiologists, only 4 of whom were musculoskeletal specialists. None of the images were checked by a second radiologist to confirm the result. This difference in level of radiologist training and experience may explain some of the reduced sensitivity and reflects practice in a standard hospital. This is consistent with work previously published by Theodoropoulos et al. (2010). Sample populations from previous studies included normal shoulders, but our study only included patients who had clinical symptoms severe enough to warrant a surgical intervention. These patients were therefore more likely to have pathology when examined at arthroscopy.

Our findings highlight the importance of an accurate history and a clinical examination by a specialist shoulder surgeon in the management of glenohumeral instability. Indeed, in these hands, the need for costly investigations such as MRA might be reduced and the pathway of the patient might be made faster and more efficient.

## References

[CIT0001] Bankart A SB (1923). Recurrent or habitual dislocation of the shoulder-joint. Br Med J.

[CIT0002] Beltran J, Bencardino J, Mellado J, Rosenberg ZS, Irish RD (1997). MR arthrography of the shoulder: variants and pitfalls. Radiographics.

[CIT0003] Chandnani VP, Yeager TD, DeBerardino T, Christensen K, Gagliardi JA (1993). Glenoid labral tears: prospective evaluation with MR imaging, MR arthrography, and CT arthrography. AJR Am J Roentgenol.

[CIT0004] Flannigan B, Kursunoglu-Brahme S, Snyder S, Karzel R, Del Pizzo W, Resnick D (1990). MR arthrography of the shoulder: comparison with conventional MR imaging. AJR Am J Roentgenol.

[CIT0005] Hobby J, Griffin D, Dunbar M, Boileau P (2007). Is arthroscopic surgery for stabilisation of chronic shoulder instability as effective as open surgery? A systematic review and meta-analysis of 62 studies including 3044 arthroscopic operations. J Bone Joint Surg (Br).

[CIT0006] Holzapfel K, Waldt S, Bruegel M, Paul J, Heinrich P, Imhoff AB, Rummeny EJ, Woertler K (2010). Inter- and intraobserver variability of MR arthrography in the detection and classification of superior labral anterior posterior (SLAP) lesions: evaluation in 78 cases with arthroscopic correlation. Eur Radiol.

[CIT0007] Iqbal HJ, Rani S, Mahmood A, Brownson P, Aniq H (2010). Diagnostic value of MR arthrogram in SLAP lesions of the shoulder. Surgeon.

[CIT0008] Magee T (2009). 3-T MRI of the shoulder: Is MR Arthrography necessary?. AJR.

[CIT0009] Magee T, Williams D (2006). Sensitivity and specificity in detection of labral tears with 3.0 T MRI of the shoulder. AJR.

[CIT0010] Newberg AH, Munn CS, Robbins AH (1985). Complications of arthrography. Radiology.

[CIT0011] Palmer WE, Brown JH, Rosenthal DJ (1994). Labral ligamentous complex of the shoulder: evaluation with MR arthrography. Radiology.

[CIT0012] Palmer WE, Caslowitz PL (1995). Anterior shoulder instability: diagnostic criteria determined from prospective analysis of 121 MR arthrograms. Radiology.

[CIT0013] Palmer WE, Brown JH, Rosenthal DJ (1994). Labral ligamentous complex of the shoulder: evaluation with MR arthrography. Radiology.

[CIT0014] Theodoropoulos JS, Andreisek G, Harvey EJ, Wolin P (2010). Magnetic resonance imaging and magnetic resonance arthrography of the shoulder: dependence on the level of training of the performing radiologist for diagnostic accuracy. Skeletal Radiol.

[CIT0015] Tirman PF, Palmer WE, Feller JF (1997). MR arthrography of the shoulder. Magn Reson Imaging Clin North Am.

[CIT0016] Waldt S, Burkart A, Imhoff AB, Bruegel M, Rummeny EJ, Woertler K (2005). Anterior shoulder instability: accuracy of MR arthrography in the classification of anteroinferior labroligamentous injuries. Radiology.

